# Outcome analysis following removal of locking plate fixation of the proximal humerus

**DOI:** 10.1186/1471-2474-9-138

**Published:** 2008-10-12

**Authors:** Chlodwig Kirchhoff, Volker Braunstein, Sonja Kirchhoff, Christoph M Sprecher, Ben Ockert, Florian Fischer, Bernd A Leidel, Peter Biberthaler

**Affiliations:** 1 Department of Orthopedic Sports Surgery, Technische Universitaet Muenchen, Connollystrasse 32, D-80809 Munich, Germany; 2 Department of Traumatology and Orthopaedic Surgery – Campus Innenstadt, Ludwig-Maximilians Universitaet, Nussbaumstrasse 20, D-80336 Munich, Germany; 3 AO Research Institute, Clavadelerstrasse 8, CH-7270, Davos, Switzerland; 4 Department of Clinical Radiology – Campus Grosshadern, Ludwig-Maximilians Universitaet, Marchioninistrasse 15, D-81373 Munich, Germany; 5 Institute of Forensic Medicine Ludwig-Maximilians Universitaet, Nussbaumstrasse 26, D-80336 Munich, Germany

## Abstract

**Background:**

Concerning surgical management experience with locking plates for proximal humeral fractures has been described with promising results. Though, distinct hardware related complaints after fracture union are reported. Information concerning the outcome after removal of hardware from the proximal humerus is lacking and most studies on hardware removal are focused on the lower extremity. Therefore the aim of this study was to analyze the functional short-term outcome following removal of locking plate fixation of the proximal humerus.

**Methods:**

Patients undergoing removal of a locking plate of the proximal humerus were prospectively followed. Patients were subdivided into the following groups: Group HI: symptoms of hardware related subacromial impingement, Group RD: persisting rotation deficit, Group RQ: patients with request for a hardware removal. The clinical (Constant-Murley score) and radiologic (AP and axial view) follow-up took place three and six months after the operation. To evaluate subjective results, the Medical Outcomes Study Short Form-36 (SF-36), was completed.

**Results:**

59 patients were included. The mean length of time with the hardware in place was 15.2 ± 3.81 months. The mean of the adjusted overall Constant score before hardware removal was 66.2 ± 25.2% and increased significantly to 73.1 ± 22.5% after 3 months; and to 84.3 ± 20.6% after 6 months (p < 0.001). The mean of preoperative pain on the VAS-scale before hardware removal was 5.2 ± 2.9, after 6 months pain in all groups decreased significantly (p < 0.001). The SF-36 physical component score revealed a significant overall improvement in both genders (p < 0.001) at six months.

**Conclusion:**

A significant improvement of clinical outcome following removal was found. However, a general recommendation for hardware removal is not justified, as the risk of an anew surgical and anesthetic procedure with all possible complications has to be carefully taken into account. However, for patients with distinct symptoms it might be justified.

## Background

Fractures of the proximal humerus are the third most common type of fracture [1]. A 3-fold increase in osteoporosis-related fractures is expected in the year 2030 [2,3].

Although the appropriate management of displaced or unstable proximal humeral fractures remains a controversial, open reduction and internal fixation (ORIF) using fixed-angle interlocking plate seems to be a promising alternative [1,4]. The proximal humerus internal locking system (Philos^®^; Synthes^®^, Oberdorf, Switzerland) consists of a low-profile plate with proximal locking screws allowing for the fixation of fractures previously considered to be inoperable or treatable only with arthroplasty [5].

Though several authors report distinct hardware related problems, such as pain, impingement or movement deficits [5-8]. Pain, tissue irritation or impaired function after fracture union as well as patient's request are typical indications for implant removal in orthopedic practice [9,10]. While these procedures are frequently considered as simple, they can be challenging at the same time [11,12]. Furthermore, hardware removal can lead to additional pain and complications, such as neurovascular injury, re-fracture or recurrence of deformity [13]. Although implant removal contributes to up to 30% of all elective orthopedic procedures, only few studies on outcome and complications of hardware removal exist [14,15]. Furthermore, these studies have only been conducted in a retrospective manner; according to our knowledge there has been no controlled prospective studies on hardware removal around the shoulder joint.

Therefore the aim of this study was to prospectively analyze whether the removal of locking plate fixation of the proximal humerus improves the patients clinical outcome.

## Methods

From July 2003 to August 2007, all patients undergoing an elective removal of a locking plate after ORIF of a proximal humeral fracture were enrolled in this prospective follow-up study. The study was approved by the ethics committee at Ludwig-Maximilians University (reference number: 264/06). Written informed consent was obtained from each patient. Inclusion criteria were as follows: displaced proximal humeral fracture, as defined by Neer criteria as a displaced fracture with displacement of >1.0 cm or 45° angulation, treated initially with a locking plate (Philos^®^; Synthes^®^, Oberdorf, Switzerland). Postoperatively, all patients underwent a standardized physiotherapeutic program, starting on the first day. In general, full active motion was allowed after the first radiologic follow-up 6 weeks after surgery. Permission to increase force transmission was given 12 weeks postoperatively. Patients were subsequently reevaluated at 6, 12, and 18 weeks, and 6 and 12 months. The indication for hardware removal was stated earliest 12 month after initial ORIF. Radiographs were taken before hardware removal. Union of the fracture was defined as radiographic presence of mature callus in two planes as determined by an experienced musculoskeletal radiologist.

Patients in whom hardware was removed due to known infection, secondary screw perforation or avascular necrosis (AVN) of the humeral head and subsequent prosthetic replacement were excluded from the study. According to the reason for hardware removal, the patients were divided into three subgroups: Group HI: symptoms of hardware related subacromial impingement due to superior plate placement. A distance <8 mm from the proximal end of the plate to the upper margin of the greater tuberosity was defined as superior placement following the AO-instructions for the implant. Subacromial impingement was stated if the following clinical criteria were positive: 1^st ^painful arc 60°-120° and 2^nd ^a positive Neer's sign and 3^rd ^a positive Hawkins/Kennedy test. Group RD: persisting significant external rotation deficit of <15° and Group RQ: patients with the explicit request for a hardware removal. The baseline data in terms of patients' age, sex, medical comorbidities, duration of fixation device implantation, Constant-Murley (Constant) score and average pain using the Visual Analog Scale (VAS: ranging from 0: no pain to 10: worst imaginable pain) were recorded preoperatively [16,17]. To evaluate the patients' subjective results, the Medical Outcomes Study Short Form-36 (SF-36), a standardized survey assessing functional outcome, was also completed preoperatively [18]. The SF-36 is a self-administered generic questionnaire designed to evaluate health-related quality of life. The instrument measures eight health domains using eight scales assessing physical function (PF), role limitation due to physical problems (RP), body pain (BP), general health (GH), vitality (VT), social function (SF), role limitation due to emotional problems (RE), and mental health (MH). The subscales range from 0–100; low scores imply poor health status and high scores correspond to a good health status.

### Surgical technique

In all cases an experienced orthopedic surgeon performed the surgery with the patient laying in a 'beach chair' position under general anesthesia. The initial delto-pectoral approach was used with minimal soft-tissue dissection. The plate was exposed, and then first the tension band wiring (FiberWire^®^, Arthrex^®^, Naples, USA) was removed. After removal of all screws the plate was taken out. Complete hardware removal was confirmed by fluoroscopy. If necessary, exostoses around the former plate bed were debrided. Patients of group RD received an additional open arthrolysis of the joint. The wound was closed over a suction drain, which was removed after 24 hours. Postoperatively the arm was supported in a sling for one day. Physiotherapy with full range of motion and weight bearing was started on the first day post-surgery with duration of six weeks.

### Follow-up

The clinical (Constant score), assessment of pain (VAS) and radiologic (true-ap and axial view) follow-up was performed three and six months after the operation. Constant score values were normalized based on age and sex. To evaluate subjective results, the SF-36 was completed at six months post-surgery. The SF-36 results were compared to an age and sex-matched German normative population [19]. At six months, a patient's satisfaction questionnaire was handed out to the patients. It consisted of three questions: (1) are you happy that the hardware was removed? (2) Would you have the surgery performed again? (3) Do you think your overall function has improved since you underwent this procedure?

### Statistical Analysis

Data are given as mean ± standard deviation (SD). The results of the different groups were compared for each indicator using the Mann-Whitney U test. For assessing differences between the different time points univariate analysis of variance with Tukey post hoc procedure for multiple comparisons was used. The level of significance was set at p < 0.05. Statistical analysis was performed using Sigma Stat 3.1 software (Systat^® ^Inc, Chicago, IL, USA).

## Results

From July 2003 to August 2007 a total number of 282 proximal humeral fractures in 277 patients has been seen at our department. From these, 231 fractures in 226 patients have been treated by ORIF. In total 79 patients underwent plate removal. Out of these 79 patients 20 had to be excluded due to known infection, secondary screw perforation or AVN of the humeral head and subsequent prosthetic replacement. So, in total 59 patients (30 women, 29 men) with a mean age of 55 ± 14 yrs were enrolled. The right upper limb was affected in 30 patients (50.8%), the left in 29 (49.2%). 15 patients were retired or out of work (25.4%), and 44 patients were active (74.6%) preoperatively. The indication for the initial operation was based on the Neer description of a displaced fracture and determined by an experienced orthopedic surgeon based on plain radiographs. In addition, the fractures were classified using the Arbeitsgemeinschaft Fuer Osteosynthesefragen (AO)/Association for the Study of Internal Fixation (ASIF) classification of proximal humerus fractures. Group distribution of fracture type was as follows: 11 patients (18.6%) had a type A-, 31 (52.5%) had a B- and 17 (28.8%) patients had a type C-fracture. Group distribution by Neer segment classification was as follows: 18 (30.5%) patients with 2-part-, 33 (55.9%) with 3-part- and 8 (13.6%) patients with 4-part fractures. The mean duration of fixation device implantation was 15.2 ± 3.8 months. The distribution according to reasons for hardware removal was as follows: Group HI (hardware related impingement due to superior plate) of 25 (42.4%) patients, Group RD (rotation deficit) of 13 (22.0%) patients, and Group RQ (patient's request) consisting of 21 (35.6%) patients.

### Functional outcome

**Figure 1:**
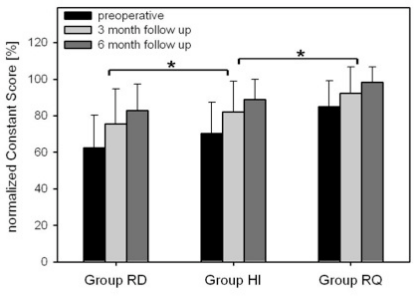
**Functional outcome assessed by normalized Constant Score preoperative, 3 and 6 month after removal of PHILOS plate.** Data are given as mean ± SD, * p-values < 0.05 comparing preoperative results to 3- and 6-month follow-up data. ANOVA on ranks followed by Tukey. Abbreviations: RD, Rotation Deficit; HI, Hardware related Impingement; RQ, Patients Request.

Regarding symptoms of impingement after 6 months in group HI 21 patients (84%) had no residuals at all, 4 patients (16%) had an isolated positive Hawkins-Kennedy test. The mean adjusted overall Constant score before hardware removal was 66.2 ± 25.2%. A significant increase to 73.1 ± 22.5% was found at 3 months and to 84.3 ± 20.6% at 6 months (p < 0.001) after removal. Analysis of the three different groups revealed significant differences (p < 0.001) in the preoperative Constant score with group RD at 62.5 ± 18.8%, group HI at 75.6 ± 16% and group RQ at 82.9 ± 14.4%. After 3 months the Constant score increased significantly in all three groups to 70.4 ± 16.9% for group RD, to 82.2.6 ± 16.7% for group HI and to 88.8 ± 11.0% for Group RQ. Finally, after 6 months, the Constant score of all group increased significantly as well (p < 0.001). Patients of group RD presented with the lowest score of 84.9 ± 14.3% followed by group HI with a score of 92.3 ± 14.3% and group RQ with 98.2 ± 8.4%. Group RD revealed a score of 3.1 ± 1.8 (maximum of 10 points) for external rotation before plate removal, 8.4 ± 2.5 at 3 months and 8.5 ± 2.8 at 6 months after removal, thereby showing a significant improvement in external rotation (p < 0.001). Age-related results are summarized in Fig. 1.

### Pain

**Figure 2:**
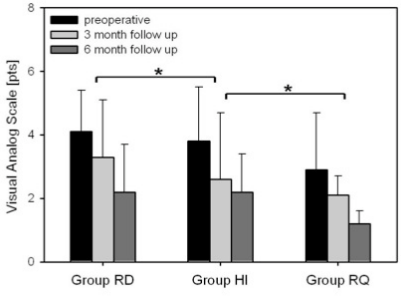
**Pain assessed by Visual Analog Scale preoperative, 3 and 6 month after removal of PHILOS plate. **Data are given as mean ± SD, * p-values <0.001 comparing preoperative results to 3- and 6-month follow-up data. ANOVA on ranks followed by Tukey. Abbreviations: RD, Rotation Deficit; HI, Hardware related Impingement; RQ, Patients Request.

The mean preoperatively assessed pain on the VAS-scale before hardware removal was 5.2 ± 2.9. Analysis of the different groups revealed significant differences (p < 0.001) in the preoperative pain level for group RD 4.1 ± 1.3, for group HI with 3.8 ± 1.7 and group RQ with 2.9 ± 1.8. After 3 months the pain level decreased significantly in all three groups to 3.3 ± 1.8 for group RD, to 2.6 ± 2.1 for group HI and to 2.1 ± 0.6 for Group RQ. After 6 months in all groups pain decreased significantly to 2.2 ± 1.5 in group RD, to 2.2 ± 1.2 for group HI and to 1.2 ± 0.4 for Group RQ (p < 0.001). Results are depicted in Fig. 2.

### Life Quality

The SF-36 physical component score revealed a significant overall improvement in both genders (p < 0.001) at six months after implant removal. The actual mean score increased from 45.8 ± 6.3 at baseline to 58.2 ± 3.5 at six months follow-up for women and from 58.7 ± 8.4 at baseline to 64.4 ± 6.2 for men. Both genders improved significantly in all sub-scales 6 months after plate removal. In comparison to norm values women reported still worse results concerning the following sub-scales: role physical (p < 0.04), bodily pain (p < 0.038), general health (p < 0.037), vitality (p < 0.051), social functioning (p < 0.02), and role emotional (p < 0.03). There was no statistical significant difference found in women at 6 months regarding physical functioning and mental health. Men reported in comparison to norm values significant worse results after six month regarding physical functioning (p < 0.02), role physical (p < 0.03), bodily pain (p < 0.04), vitality (p < 0.049), social functioning (p < 0.009) and role emotional (p < 0.01). There was no difference regarding general and mental health. For detailed SF-36 data see Table 1 and 2.

**Table 1:** SF-36 in women preoperative and 6 month after removal of PHILOS plate and the age- and gender matched norms of the German population


**SF-36 sub-scales**	**Preoperative ** (n = 30) Mean ± SD	**6-month follow-up ** (n = 30) Mean ± SD	**Norms of the German population**	* p-values	^# ^**p-values**

**Physical functioning**	45.5 ± 10.2	58.2 ± 11.8	67.9 ± 19.9	***0.04***	0.12
**Role physical**	41.4 ± 10.8	59.6 ± 11.2	63.6 ± 36.9	***0.0009***	***0.04***
**Bodily pain**	42.2 ± 8.7	60.5 ± 11.4	64.9 ± 26.7	***0.00087***	***0.038***
**General health**	38.9 ± 7.7	49.0 ± 9.7	54.8 ± 16.9	***0.048***	***0.037***
**Vitality**	42.8 ± 20.9	51.3 ± 21.8	57.5 ± 18.2	***0.042***	***0.051***
**Social functioning**	65.9 ± 17.3	79.3 ± 14.3	87.3 ± 16.9	***0.03***	***0.02***
**Role emotional**	66.8 ± 6.9	73.2 ± 9.8	83.3 ± 30.9	***0.04***	***0.03***
**Mental health**	58.9 ± 8.5	64.3 ± 8.4	72.7 ± 16.9	***0.048***	0.51

Data are given as mean ± SD, * p-values comparing patients preoperative results to 6-month follow-up data, ^# ^p-values comparing patients results at 6-month follow-up to normative data. ANOVA on ranks followed by Tukey.

* p-values comparing patients preoperative results to 6-month follow-up data.

^# ^p-values comparing patients results at 6-month follow-up to normative data

**Table 2:** SF-36 in men preoperative and 6 month after removal of PHILOS plate and the age- and gender matched norms of the German population


**SF-36 sub-scales**	**Preoperative ** (n = 29) Mean ± SD	**6-month follow-up ** (n = 30) Mean ± SD	**Norms of the German population**	* p-values	^# ^**p-values**

**Physical functioning**	52.3 ± 12.3	67.1 ± 23.6	72.9 ± 22.1	***0.04***	***0.02***
**Role physical**	44.8 ± 17.5	58.8 ± 11.2	65.7 ± 33.5	***0.045***	***0.03***
**Bodily pain**	46.9 ± 20.1	64.7 ± 9.8	70.3 ± 22.8	***0.0006***	***0.04***
**General health**	40.3 ± 9.4	52.0 ± 12.6	56.3 ± 15.9	***0.01***	0.52
**Vitality**	44.2 ± 23.7	57.5 ± 13	61.4 ± 17.3	***0.0008***	***0.049***
**Social functioning**	69.3 ± 24.8	84.6 ± 8.2	91.8 ± 10.8	***0.0001***	***0.009***
**Role emotional**	69.1 ± 14.7	77.2 ± 19.8	84.2 ± 26.9	***0.045***	***0.01***
**Mental health**	66.9 ± 17.3	71.3 ± 11.9	75.7 ± 13.9	***0.03***	0.61

Data are given as mean ± SD, * p-values comparing patients preoperative results to 6-month follow-up data, ^# ^p-values comparing patients results at 6-month follow-up to normative data. ANOVA on ranks followed by Tukey.

* p-values comparing patients preoperative results to 6-month follow-up data.

^# ^p-values comparing patients results at 6-month follow-up to normative data

### Fracture morphology

Relating the results to the AO/ASIF fracture classification, type B fractures had the highest average Constant score (90.6 ± 17.4 pts), followed by type A and C fractures (84.9 ± 18.5 and 76.3 ± 22.8 pts) after 6 months after. Concerning the Neer classification patients with 3-part fractures presented with the highest average score (92.2 ± 18.4 pts), followed by 2- and 4-part fractures (83.8 ± 18.2 and 70.6 ± 20.6 pts).

### Duration of surgery and complications

The duration of surgery was 32.3 ± 6.8 min in patients undergoing isolated hardware removal. In patients with initial head impression fractures undergoing additional diagnostic arthroscopy the duration of surgery was 48 ± 11 min.

One (1.3%) patient suffered from impaired wound healing due to a superficial infection, treated conservatively. No patient presented with pre- or postoperative clinical signs of sensory or motor function deficits.

### Patient satisfaction

Six months after removal 42 (53.2%) patients answered the satisfaction-questionnaire. All patients stated that they were satisfied; that they would have the removal done again, and that their overall function had improved after hardware removal.

## Discussion

A number of studies have analyzed the functional outcome after locking plate osteosynthesis for displaced fractures of the proximal humerus [5,11]. For the first time we demonstrate a prospective study on the outcome of removal of locking plate fixation. We found a significant improvement of the Constant score 6 months after the removal in comparison to the preoperative status. Moreover, a significant reduction of pain was observed during follow-up. Regarding physical components of the SF-36 life quality score also a significant improvement was found after 6 months in comparison to the preoperative status.

### Patients

In the presented study 59 patients were included. Concerning gender our collective is comparable to previous studies [7]. In contrast to other surveys our patient collective is slightly younger. For comparison Voigt et al. reported about 50 patients with an average age of 65 years treated with the PHILOS plate for proximal humeral fractures [11]. We monitored patients for a period of 15.2 ± 2.8 months until removal. Hente et al. reported that patients achieved the greatest degree of recovery in terms of range of motion within the first 3 months after surgery. They observed only slight amelioration in the time thereafter [1]. This is in contrast to the results of Hepp and of Fankhauser et al showing an increase in the range of motion over time until 12 months after surgery [6,8]. However, with respect to these data, the time of at least 12 months until removal seems to guarantee a maximum post-injury recovery. In all patients the delto-pectoral approach was used during the initial procedure as well as the revision. Thereby a potential bias caused by different surgical approach was minimized [9].

Specific complaints following ORIF of the proximal humerus in terms of pain, impingement syndrome or movement deficits are frequently reported in literature [5,7]. We subdivided our collective into three sub-groups according to the patients' major complaints. One group consisted of patients with hardware related subacromial impingement. In these terms Kettler et al. reported in their study a 3%-rate of superior plate positioning leading to subacromial impingement [5]. Other authors reported about a greater number of cases, for example Bartsch et al. describing a 25%-rate of overhanging plates because of the implant size [20]. Summarizing the correct plate orientation with a distance <8 mm underneath the cranial border of the greater tuberosity should be confirmed intraoperatively using x-ray control to avoid this technical failure.

In patients with persisting rotational deficits we performed additional arthrolyis, as the main reason for rotational deficits might not be the plate itself, but postoperative capsular adhesions. In this context Hepp et al. previously discussed the anatomical reasons for persisting movement deficits in comparing the anterolateral deltoid splitting and the deltopectoral approach. He concluded that persisting deficits concerning abduction and flexion might primarily be due to a structurally weakening of the muscle and concerning external rotation due to scar formation in this area. They also accused postoperative adhesion of soft tissue layers to lead to a lack of conversion of force into movement [9]. Summarizing, it seems reasonable to accuse not plate removal but arthrolysis for the significant improvement of clinical results within this group. However, the additional affect of plate removal cannot be distinguished, as there are no data on patients, receiving isolated arthrolysis without plate removal. A controlled prospective trail focusing on plate removal vs. arthrolysis is ethically not feasible.

### Functional outcome

In the present study the mean adjusted overall Constant score before hardware removal was 66.2 pts and increased significantly to 73.1 pts after 3 months; and to 84.3 after 6 months. Moonot et al. recently reported about their experience with the PHILOS locking plate and noticed a mean Constant score of 66.5 pts at an average follow-up of 11 months after initial surgery [21]. This interval is comparable to the data in the current study with a mean interval of 9.8 months until hardware removal. Also the preoperative Constant scores are consistent with these described by other authors. Bjorkenheim et al described a study of 72 patients with a mean Constant score of 72 at follow-up after six months [22]. Koukakis et al published a series of 20 patients with a mean Constant score of 76 after six months [7]. However, although all authors reported cases, in which hardware removal became necessary, the precise medical indications remain unclear. Concerning medical reasons the analysis of the three different groups in the present study revealed significant differences. In patients with persistent rotational deficits or hardware related subacromial impingement the improvement might be explicable. However, the group of patients without documented medical complaints also improved significantly from 88.8 to 98.2% after surgery, representing excellent Constant score results.

### Pain and Life Quality

In addition, we observed a significant improvement regarding the average pain (VAS) as well as the health related quality of life. The significant reduction of pain is comparable to other studies focusing on hardware removal. Regarding deep implants such as intramedullary nails, a retrospective review of eighty patients by Dodenhoff et al. noted that eleven of seventeen patients undergoing implant removal following a healed femoral fracture experienced pain relief [23]. With tibial intramedullary nails, knee pain is known as a common indicator for removal. Keating et al. showed a 45% rate of complete knee pain relief in 110 patients after tibial nail removal. In addition, 35% of the patients experienced partial relief, whereas 20% of the patients reported no relief [24]. In a retrospective review of 169 patients, Townend et al. noted that 27% had a complete pain relief and 69% had marked relief after nail removal [25]. However, 3.2% of the patients reported worsening pain after hardware removal. In a retrospective study Gosling et al. in 2004 found that after femoral nail removal in fifty-one patients who had been asymptomatic preoperatively, ten (20%) patients developed symptoms postoperatively [26].

### Limitations of the study

One limitation of our study is the unknown percentage of patients with a healed fracture who had removal of hardware elsewhere. Patients who underwent fracture fixation at our institution may have had follow-up care provided elsewhere, and some were lost to follow-up. In addition, one of the difficulties in evaluating patients for improvement of life quality on the basis of the SF-36 questionnaire is the fact that they may have additional disabilities not related to hardware that compromise the overall functional and mental scores. Furthermore, posttraumatic arthritis related to the initial injury may have developed in some patients. However, an exact determination of the joint status in this early interval after the initial injury might only be feasible arthroscopically, which was not performed in a routine manner.

## Conclusion

A significant improvement of functional outcome parameters following removal of locking osteosynthesis of the proximal humerus was found in the presented study. This knowledge might be important for counselling the patients about the expected level of success when planning the removal of fracture fixation of the proximal humerus. Although in our series only a very low rate of complications was found, a general recommendation for hardware removal is not justified. The risk of an anew surgical and anaesthetic procedure with all possible complications has to be carefully taken into account. However, following our results for patients suffering from distinct symptoms the recommendation for hardware removal is justified. Concluding, especially in active patients with residual deficits the removal of osteosynthesis of the proximal humerus might be promising.

## Competing interests

The authors declare that they have no competing interests.

## Authors' contributions

CK is responsible for the study design, wrote the manuscript. VB is responsible for the study design, and participated in patients' evaluation. SB is responsible for the radiologic investigations and helped to draft the manuscript. CS is responsible for data editing and carried out the statistical evaluations. BO carried out the follow up examinations. PB is responsible for the study design, and performed the surgeries.

## Pre-publication history

The pre-publication history for this paper can be accessed here:


